# Preoperative gastric volume assessment using ultrasound in cerebral palsy pediatric patients: a prospective observational study

**DOI:** 10.1016/j.bjane.2024.844541

**Published:** 2024-07-16

**Authors:** Jiwon Han, Hyo-Seok Na, Seihee Min, Hyun-Jung Shin

**Affiliations:** a Chung-Ang University College of Medicine, Department of Anesthesiology and Pain Medicine, Seoul, Republic of Korea; b Seoul National University Bundang Hospital, Department of Anesthesiology and Pain Medicine, Seoul, Republic of Korea; c Seoul National University College of Medicine, Department of Anesthesiology and Pain Medicine, Seoul, Republic of Korea

**Keywords:** Cerebral palsy, Child, Fasting, Stomach, Ultrasonography

## Abstract

**Background:**

Although cerebral palsy is a risk factor for aspiration, there is insufficient research on residual gastric volume after preoperative fasting in children with cerebral palsy. We evaluated the incidence of a full stomach by ultrasound assessment of the gastric volume in children with cerebral palsy who underwent orthopedic surgery after preoperative fasting.

**Methods:**

The patients fasted for 8 h for solid foods and 2 h for clear liquids. We obtained the gastric antral cross-sectional area using ultrasound in the semi-recumbent and right lateral decubitus positions. A calculated stomach volume > 1.5 mL.kg^−1^ was considered as full, which poses a high aspiration risk. The primary outcome was the incidence of full stomach, and the secondary outcomes were the qualitative gastric volume, correlation of disease severity categorized according to the Gross Motor Function Classification System with the residual gastric volume, gastric volume per body weight, and qualitative gastric volume.

**Results:**

Thirty-seven pediatric patients with cerebral palsy, scheduled for elective orthopedic surgery, were included for analysis. Full-stomach status was observed in none, and the gastric volume per body weight was 0.5 (0.4–0.7) mL.kg^−1^. No significant differences were observed in the residual gastric volume (*p* = 0.114), gastric volume per body weight (*p* = 0.117), or qualitative grade of gastric volume (*p* = 0.642) in relation to disease severities.

**Conclusion:**

Children with cerebral palsy who fasted preoperatively had empty or nearly empty stomachs. Further studies are required to determine the optimal fasting duration for such children.

## Introduction

The European Society of Anaesthesiology and Intensive Care^1^ introduced preoperative fasting guidelines for healthy children that encourage the consumption of clear fluids for up to 1 hour and allow solid food intake until 6 hours before anesthesia induction. However, their generalization for children with comorbidities requires caution because of limited data.

Cerebral Palsy (CP) is a neurological disease accompanied by motor dysfunction caused by damage to the central nervous system. Depending on the degree of damage, CP is characterized by sensory, perceptual, hearing, visual, language, and cognitive impairments, which are accompanied by various clinical features ranging from mild coordination disorders to bed-ridden status.^2^ In addition, most patients (92%) present with gastrointestinal motility dysfunctions such as regurgitation, vomiting, abdominal pain, constipation, delayed gastric emptying, and abnormal esophageal motility, which increase the risk of pulmonary aspiration.^3^ Particularly, 41% of pediatric patients with CP experience chronic pulmonary aspiration.^3^

Adequate preoperative fasting is required to prevent pulmonary aspiration of gastric contents.^4^ However, excessive fasting may also lead to hypoglycemia and dehydration.^4^ Children are particularly more vulnerable to these problems than adults. Furthermore, only a few studies have reported on the residual gastric volume after fasting in children with CP. To improve their perioperative care, clinicians should determine the appropriate fasting time.

We hypothesized that preoperative residual gastric volume, classified as a high-risk factor for pulmonary aspiration, may be larger in children with CP than that in the general population. Thus, we aimed to evaluate the incidence of a full stomach by assessing the gastric volume using ultrasound in children with CP who underwent orthopedic surgery after fasting.

## Methods

### Study design, ethics, and participants

This prospective observational study was conducted between June 2021 and February 2022. It was approved by the Institutional Review Board of Seoul National University Bundang Hospital (approval number: B-2104-681-301), and the study protocol was registered at ClinicalTrials.gov (NCT05185336). Written consent was obtained from the parents of all included patients. Consent was also obtained from patients if they could write and understand the study protocol.

Pediatric patients with CP (5–18 years) scheduled for elective orthopedic surgery under general anesthesia were eligible for inclusion. Patients with gastroesophageal reflux disease, a history of esophageal or stomach surgery, or a nasogastric or gastrostomy tube for feeding were excluded. We retrieved information on patient demographics, comorbidities, and medications.

### Preoperative fasting guidelines

At our institution, preoperative fasting time for pediatric patients is 8 hours for solid foods and 2 hours for clear liquids.

### Ultrasound scanning for gastric volume evaluation

In the preoperative holding area, the Cross-Sectional Area (CSA) of the gastric antrum was measured in the semi-recumbent and Right Lateral Decubitus (RLD) positions^5^ using a low-frequency convex array transducer (rC60xi, 2‒5 Hz) connected to an ultrasound system (SonoSite Edge II; FUSIFILM Sonosite Inc., Bothell, Washington, United States). Ultrasonographic measurement of CSA was performed by an experienced anesthesiologist according to the standard scanning protocol.^6^ The gastric antrum was identified at the epigastric level in the sagittal or parasagittal view between the left lobe of the liver and aorta or inferior vena cava.

The CSA was calculated using the Perpendicular Anteroposterior (AP) and Craniocaudal (CC) diameters of the gastric antrum as follows: *CSA* (*cm*^2^) = π × *AP* × *CC*/4. The following formula was used to estimate the gastric volume with antral CSA in the RLD position in pediatric patients: gastricvolume=−7.8+(3.5×RLDCSA)+0.127×age(months).^7^ A full stomach was defined as a measured gastric volume > 1.5 mL.kg^−1^ or the presence of solid content in the stomach.^8^ A calculated value < 1 mL was considered 0 mL.

In addition, we performed a qualitative assessment that determined the risk of pulmonary aspiration based on the gastric contents according to change in position as follows:^9^ Grade 0, no gastric contents are visible in the antrum in both the semi-recumbent and RLD positions; Grade 1, gastric contents are visible only in the RLD position and not in the semi-recumbent position; and Grade 2, gastric contents are visible in both positions.

### Outcome measurements

The primary outcome was the incidence of full stomach. The secondary outcomes included the qualitative grade of gastric volume and the correlation between CP severity and the residual gastric volume, gastric volume per body weight, and qualitative grade of gastric volume. CP severity was determined using a Gross Motor Function Classification System (GMFCS) as follows:^10^ Level 1, walks without restriction, limitations in more advanced gross motor skills; Level 2, walks without restrictions, limitations in walking outdoors and in the community; Level 3, walks with assistive mobility devices, limitations in walking outdoors and in the community; Level 4, self-mobility with limitations, children are transported or use power mobility outdoors and in the community; and Level 5, severely limited self-mobility even with the use of assistive technology. Additionally, we evaluated the adverse effects of fasting such as signs of dehydration and hypoglycemia (tachycardia, hypotension, dry mouth, and sweating).

### Sample size

We calculated the sample size using a web-based system (https://clincalc.com/stats/samplesize.aspx). In a previous study,^8^ the incidence of full stomach was 6.2% in the general population after preoperative fasting, and another study^11^ reported that the normogastria incidence of pediatric patients with CP was 80%. Assuming the incidence of a full stomach in pediatric patients with CP to be 20%, we determined 34 participants with an alpha level of 0.05 and power of 80% to obtain a statistically significant difference. Considering a dropout rate of 10%, 38 participants were required for this study.

### Statistical analysis

The Shapiro-Wilk test was performed to determine the normality of continuous variables. Normally distributed data are expressed as mean (standard deviation). For skewed data, the results are presented as medians (interquartile ranges). The Kruskal-Wallis test was performed to analyze continuous data (CSA, gastric volume, and gastric volume per weight). Categorical data (qualitative grade) were analyzed using Fisher's exact test, and the results are expressed as numbers (proportions). We calculated Spearman's rank correlation coefficients to examine the severity of CP, residual gastric volume, residual gastric volume per body weight, and qualitative grade of gastric volume. Statistical Package for Social Sciences (SPSS; ver. 27; IBM Corp., Armonk, NY, USA) software was used for data analysis, and all *p-*values < 0.05 were considered significant.

## Results

**Figure 1:**
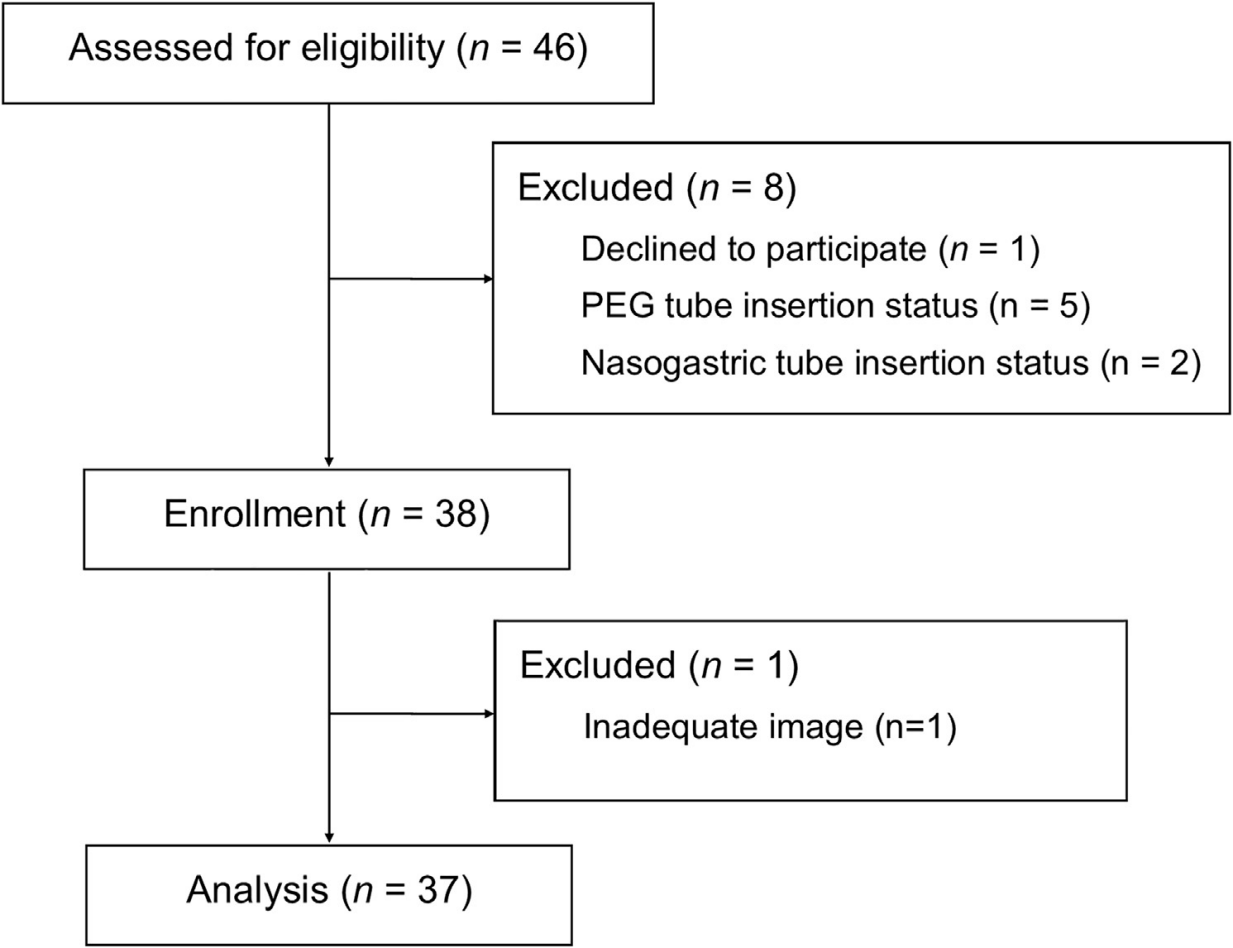
Flow diagram for patient selection. PEG, Percutaneous Endoscopic Gastrostomy.

Forty-six patients were evaluated for eligibility. Eight of them were excluded for the following reasons: one refused to participate, and seven were supplied with nutrition through nasogastric or gastrostomy tubes. One patient was excluded from the final analysis due to unsuitable ultrasound images. Finally, thirty-seven patients were included for analysis (Fig. 1). Table 1 summarizes the baseline patient characteristics.

**Table 1:** Patient characteristics.

Variables	n = 37
Age (years)	11 (3.4)
Height (cm)	138 (18)
Weight (kg)	38.2 (14.7)
ASA physical status	
II	33 (89.2%)
III	4 (10.8%)
Comorbidities	
Cardiac abnormality	3 (8.1%)
Epilepsy	4 (10.8%)
Medications	
Anticonvulsants	9 (24.3%)
Laxatives	4 (10.8%)
Prokinetic agents	2 (5.4%)
Respiratory medications	2 (5.4%)
Others	2 (5.4%)

Data are expressed as mean (standard deviation) or number (proportion).

ASA, American Society of Anesthesiologists.

Full-stomach status was not observed in any patient. The median gastric volume was 20.9 (13.4–25.4) mL and the median gastric volume per body weight was 0.5 (0.4–0.7) mL.kg^−1^ (Table 2). Table 2 also shows the gastric volume according to the CP level. We observed no significant differences in the residual gastric volume (*p* = 0.114), gastric volume per body weight (*p* = 0.117), or qualitative grade of gastric volume (*p* = 0.642) among the GMFCS grades. None of the patients were classified as Level 5.

**Table 2:** Gastric volume according to the severity of cerebral palsy categorized by GMFCS.

	Total	GMFCS level					*p-*value
		1	2	3	4	5	
	(n = 37)	(n = 3)	(n = 11)	(n = 6)	(n = 17)	(n = 0)	
**Quantitative analysis**							
Gastric volume (mL)	20.9 (13.4‒25.5)	18.5 (15.3‒20.4)	16.9 (11.6‒20.9)	25.9 (14.8‒141.1)	22.1 (15.4‒26.0)	NA	0.114
Gastric volume (mL.kg^−1^)	0.5 (0.4‒0.7)	0.6 (0.5‒0.6)	0.4 (0.3‒0.6)	0.6 (0.5‒0.9)	0.5 (0.4‒0.8)	NA	0.117
CSA (RLD, cm^2^)	2.7 (1.8‒4.0)	2.8 (2.6‒3.5)	2.2 (1.7‒3.3)	3.3 (2.1‒6.6)	3.0 (1.7‒4.0)	NA	0.527
**Qualitative assessment**							
Grade 0, n (%)	29 (78.4%)	3 (100%)	8 (72.7%)	4 (66.7%)	14 (82.4%)	NA	0.642
Grade 1, n (%)	8 (21.6%)	0 (0%)	3 (27.3%)	2 (33.3%)	3 (17.6%)	NA	
Grade 2, n (%)	0 (0%)	0 (0%)	0 (0%)	0 (0%)	0 (0%)	NA	

Data are expressed as median (interquartile range) or number (proportion).

CSA, Cross-Sectional Area; GMFCS, Gross Motor Function Classification System; RLD, Right Lateral Decubitus; NA, Not Applicable.

No significant correlations were observed between CP severity and residual gastric volume, residual gastric volume per body weight, or qualitative grade of gastric volume (Table 3). Additionally, there were no cases of adverse events attributed to fasting.

**Table 3:** Spearman's rank correlation coefficients between the severity of cerebral palsy according to GMFCS and residual gastric volume (n = 37).

	GMFCS	*p-*value
Residual gastric volume	0.305	0.066
Residual gastric volume per body weight	0.266	0.111
Qualitative grades of gastric contents	−0.020	0.908

GMFCS, Gross Motor Function Classification System.

## Discussion

In this study, we evaluated the preoperative residual gastric volume in children with CP and observed no patients with full-stomach status after preoperative fasting. In addition, we observed no correlation between CP severity and residual gastric volume. These findings provide evidence for the safety of fasting and warrant further studies to determine an optimal fasting time for children with CP to avoid metabolic deterioration caused by excessive fasting.

Homeostasis of gastrointestinal functions is maintained not only by the autonomic nervous system but also by the central nervous system. Predominantly, it exerts inhibitory effects on gastrointestinal motility and mucosal secretion and regulates gastrointestinal blood flow via vasoconstriction.^12^ The upper gastrointestinal tract (esophagus and stomach) is particularly more dependent on extrinsic neural input from the central nervous system than the lower gastrointestinal tract.^13^ Consequently, patients with CP experience vomiting, regurgitation, feeding difficulties, malnutrition, and aspiration.^14^ Of these symptoms, aspiration causes pneumonitis and pneumonia, which are the major causes of longer hospitalization and mortality in patients with CP.^15^^,^^16^

Patients were fasted preoperatively to prevent aspiration. Aspiration is defined as the inhalation of oropharyngeal or gastric contents into the lower respiratory tract, leading to conditions such as bronchospasm, hypoxia, pneumonitis, pneumonia, and acute respiratory distress syndrome.^17^^,^^18^ Aspiration pneumonia occurs when the aspirated material causes an infectious process; it affects approximately 1% of adult surgical patients and is associated with increased rates of intensive care unit admission, hospitalization duration, medical costs, and in-hospital mortality.^17^ Furthermore, aspiration pneumonia accounts for 9% of anesthesia-related mortality.^19^ In children, the risk of aspiration is higher because of anatomical vulnerabilities, such as a small oral cavity, short laryngeal and vocal fold lengths, higher-located epiglottis tip, immature airway protective reflexes, and oropharyngeal discoordination. Patients with anatomical deformities, such as cleft palate or esophageal atresia, as well as those with neuromuscular diseases such as CP or myotonic dystrophy, are at a higher risk.^18^^,^^20^

The preoperative fasting guidelines for children report on the usefulness of preoperative ultrasound gastric assessment for the residual gastric volume evaluation.^1^ Gastric ultrasound is a noninvasive, real-time, inexpensive, and effective tool for evaluating stomach contents; therefore, it is central to determine the timing of the procedure and induction technique (rapid sequence vs. routine inhalation or intravenous) in non-elective surgery or aspiration in high-risk patients (hypertrophic pyloric stenosis).^21^^,^^22^ In healthy pediatric patients, ultrasound gastric examination indicated a full stomach in < 1% of the patients following the 2017 American Society of Anesthesiologists (ASA) fasting guidelines (2 h of clear fluid fasting; 6 h of solid fasting).^4^ However, it is unclear whether these fasting guidelines are safe for high-risk patients. In these cases, clinicians can use gastric ultrasound to evaluate the stomach volume and reduce the risk of aspiration. Further research is required on the safety of applying the fasting guidelines to patients with CP and for determining the optimal fasting time.

This observational study had some limitations. First, prolonged fasting durations (> 6 h) may have had an impact on stomach emptying. Well-designed research is required to determine the optimal fasting duration for patients with CP. Second, the effect of clear fluid ingestion before 1–2 h on gastric volume is unknown because all patients underwent fasting for at least 8 h for solid foods and for at least 2 h for clear liquids. This warrants additional studies that apply the current preoperative fasting guidelines. Third, the risk of aspiration increases with CP severity.^23^ However, we did not observe this correlation because of the relatively small sample size, and the patients in GMFCS 5 level were excluded because they were on nasogastric or gastrostomy feeding. Fourth, the age of the participants in this study ranged between 5 and 18 years, and the rapid physiological changes that occur in growing children may have influenced the results. Despite these limitations, this study adds to our knowledge on the safety of 8 h solid food, 2 h clear liquid fasting in children with CP.

## Conclusions

In conclusion, children with CP who fasted for 8 h for solid foods and 2 h for clear liquids had empty or nearly empty stomachs. Further studies are required to determine the optimal fasting duration for such children.

## Conflicts of interest

The authors declare no conflicts of interest.

## Appendix Supplementary materials

Image, application 1
